# Cardioprotective Mechanisms against Reperfusion Injury in Acute Myocardial Infarction: Targeting Angiotensin II Receptors

**DOI:** 10.3390/biomedicines11010017

**Published:** 2022-12-22

**Authors:** Gabriel Méndez-Valdés, Vicente Pérez-Carreño, Maria Chiara Bragato, Malthe Hundahl, Silvia Chichiarelli, Luciano Saso, Ramón Rodrigo

**Affiliations:** 1 Molecular and Clinical Pharmacology Program, Faculty of Medicine, Campus Norte, Institute of Biomedical Sciences, University of Chile, Avda. Independencia 1027, Santiago 8380000, Chile; 2 Department of Biomedical Sciences, Humanitas University, Via Rita Levi Montalcini 4, 20090 Milan, Italy; 3 Department of Biomedicine, Aarhus University, Høegh-Guldbergs Gade 10, C. F. Møllers Allé 6, 8000 Aarhus C, Denmark; 4 Department of Biochemical Sciences “A. Rossi-Fanelli”, Sapienza University of Rome, 00185 Rome, Italy; 5 Department of Physiology and Pharmacology “Vittorio Erspamer”, Faculty of Pharmacy and Medicine Sapienza University, P.le Aldo Moro 5, 00185 Rome, Italy

**Keywords:** reperfusion injury, oxidative stress, ARBs, AT1R, AT2R, angiotensin, myocardial ischemia–reperfusion injury, intracellular renin angiotensin system

## Abstract

Ischemia/reperfusion injury is a process associated with cardiologic interventions, such as percutaneous coronary angioplasty after an acute myocardial infarction. Blood flow restoration causes a quick burst of reactive oxygen species (ROS), which generates multiple organelle damage, leading to the activation of cell death pathways. Therefore, the intervention contributes to a greater necrotic zone, thus increasing the risk of cardiovascular complications. A major cardiovascular ROS source in this setting is the activation of multiple NADPH oxidases, which could result via the occupancy of type 1 angiotensin II receptors (AT1R); hence, the renin angiotensin system (RAS) is associated with the generation of ROS during reperfusion. In addition, ROS can promote the expression of NF-κΒ, a proinflammatory transcription factor. Recent studies have described an intracellular RAS pathway that is associated with increased intramitochondrial ROS through the action of isoform NOX4 of NADPH oxidase, thereby contributing to mitochondrial dysfunction. On the other hand, the angiotensin II/ angiotensin type 2 receptor (Ang II/AT2R) axis exerts its effects by counter-modulating the action of AT1R, by activating endothelial nitric oxide synthase (eNOS) and stimulating cardioprotective pathways such as akt. The aim of this review is to discuss the possible use of AT1R blockers to hamper both the Ang II/AT1R axis and the associated ROS burst. Moreover; we suggest that AT1R antagonist drugs should act synergistically with other cardioprotective agents, such as ascorbic acid, N-acetylcysteine and deferoxamine, leading to an enhanced reduction in the reperfusion injury. This therapy is currently being tested in our laboratory and has shown promising outcomes in experimental studies.

## 1. Introduction

Coronary heart disease is the leading cause of death worldwide [1]. When a patient presents with an acute myocardial infarction (AMI), the most effective therapeutic strategy to improve clinical outcomes is blood flow restoration through percutaneous coronary intervention (PCI), with or without prior thrombolysis [2]. However, this treatment has also been associated with a type of myocardial injury known as reperfusion injury [3], a process associated with an increase in the infarct size [2], where viable cardiomyocytes die due to the activation of specific signaling pathways. Studies in animal models suggest that reperfusion injury could account for up to 50% of the final infarct size [2]. Likewise, this event is associated with complications such as myocardial stunning, reperfusion arrhythmias, the non-reflux phenomenon and diastolic dysfunction [4].

Reperfusion damage is a consequence of blood flow restoration in the tissue affected by ischemia. After PCI, the rapid and massive production of reactive oxygen species (ROS) occurs, affecting the protein conformation, enzymatic activity, ligand binding and protein–protein interactions, inducing inflammation and damaging organelles along with biomolecules crucial for cell viability [4]. This ROS burst triggers different cell death pathways, such as apoptosis, necrosis or ferroptosis [2,4].

Reactive oxygen species are mainly generated in the mitochondria, where the most important dysfunction is that of the electron transport chain, followed by nicotinamide adenine dinucleotide (NADPH) oxidases [5]. One of the main agents related to the increase in ROS in cardiomyocytes is the RAS [6]. It has a significant role in the regulation of blood pressure and hydro-electrolyte homeostasis. The renin angiotensin system can be found in circulating form (circulating RAS −25%) or synthesized from local tissues (paracrine RAS −75%). The latter has its main effect in different locations, including the cardiovascular tissue [7], and it can exert an intracrine effect (intracellular RAS) [8].

Angiotensin II (Ang II) is a central multifunctional hormone of RAS that exerts its action mainly through two G protein-coupled receptors: type 1 (AT1R) and type 2 (AT2R) [8]. The angiotensin II type 1 receptor leads to prooxidant and proinflammatory activity associated with increased ROS production, vasoconstriction and cardiac remodeling, ending in cardiac hypertrophy [9]. In contrast, AT2R exerts antioxidant and anti-inflammatory activity [8]. The latter receptor modulates and counterbalances AT1R’s action by promoting vasodilation via nitric oxide (NO), reducing ROS and inflammation [8]. Interestingly, some experimental studies have shown that the application of type 1 Ang II receptor antagonists (ARBs) can reduce the size of the infarcted area in a dose-dependent way [10]. 

It has been suggested that Ang II, through the induction of moderate amounts of ROS and protein kinase C epsilon (PKCε) activation, can be involved in ischemic preconditioning (IPC) in Langendorff models. This mechanism of Ang II occurs through the activation of both AT1R and AT2R. Since IPC and Ang II share common signaling pathways at a mitochondrial level, such as the mitogen-activated protein kinase (MAPK) and NADPH oxidase pathway, it is expected that the use of IPC together with Ang II-induced preconditioning (APC) would have synergistic effects in reducing the infarct zone size [11]. 

Our hypothesis is that the administration of ARBs before reperfusion reduces the size of the infarcted area, and if it is combined with nonspecific antioxidant therapy consisting of ascorbic acid, deferoxamine (DFO) and N-acetylcysteine (NAC), it could have synergistic effects.

## 2. Angiotensin Axis

The use of ARBs as a pharmacological therapy has a substantial clinical benefit because of their action on the RAS system. Through the inhibition of the angiotensin-converting enzyme (ACE) and selective antagonism of Ang II receptors, its main use is the reduction of blood pressure in patients with arterial hypertension. The selective antagonism of the angiotensin II receptor not only allows the control of blood pressure, but it also has a complex effect on the heart and kidneys. Indeed, ARBs guarantee protection against the local effects of high blood pressure, thus avoiding the remodeling of vital structures such as the blood vessels, heart, kidneys and brain.

In accordance with this information, several studies have demonstrated that the consequences of ARB usage lead to protection against several disease states, including systolic dysfunction, systolic dysfunction after myocardial infarction (MI) and diabetic nephropathy [12].

Typically, ARBs are administered as pills per os, and four to six weeks of therapy are required to achieve the full therapeutic effects. Angiotensin receptor blockers are generally well tolerated and have a low incidence of side effects. Since ARBs do not increase bradykinin levels, the incidence of angioedema and cough is lower with respect to that of angiotensin-converting enzyme inhibitors (ACEI). There are few cases where the use of ARBs is contraindicated, specifically in patients with bilateral renal artery stenosis or patients with heart failure who have hypotension, since, sometimes, ARBs can cause hypotension [13].

### 2.1. Ang II/AT1R Axis

The physiological and pathophysiological effects of the activation of the Ang II/AT1R axis are well known. Angiotensin II exerts its effect through binding to a G-protein-coupled receptor and its main role is the maintenance of renal and cardiovascular homeostasis. Other functions are the release of proinflammatory cytokines, an increase in oxidative stress, the suppression of NO synthesis and the activation of the nuclear factor kappa-light-chain enhancer of activated B cells (NF-κB). This latter function has been reported to have an important role in angiogenesis [14]. There are two types of RAS: one on a local level and the other systemic. They are independently regulated, and their alterations trigger different pathological mechanisms [15]. The activation of this receptor is regulated by Ang II levels. Acutely, increased levels of Ang II increase the activation of this receptor [16].

### 2.2. Ang II/AT2R Axis

Type 2 Ang II receptor activation is generally described as a counter-modulatory effect of AT1R activation. It increases NO synthesis both by direct stimulation and by type 2 bradykinin receptor through endogenous bradykinin output [17,18]. AT2R also enhances dephosphorylation and participates in the tyrosine kinase growth pathway and in the activation of phosphatases. This latter action leads to the decreased expression of NF-κB and cyclooxygenase 2, and the inhibition of the JAK-STAT pathway. Due to this, AT2R activation also has an anti-inflammatory effect, which results in lower levels of both proinflammatory cytokines and prostaglandins [19,20,21]. It has been described that in some pathological processes, AT2R mimics the action of AT1R, promoting effects such as vasoconstriction and hypertrophy [22]. Its expression also increases cardiac cell numbers [23,24,25,26].

### 2.3. Angiotensin 1–7/Mas Receptor Axis

Angiotensin 1–7 (Ang 1–7) has been described to have a wide range of effects at a cardiovascular level through the activation of the Mas receptor (MasR). In cardiomyocytes, acute exposure to Ang 1–7 stimulates NO release by activating eNOS and neuronal nitric oxide synthase (nNOS) [27,28]. In addition, it has been seen that in animal models, it has different cardioprotective effects; among them are a reduction in the generation of ischemia–reperfusion-induced arrhythmias and the improvement of post-ischemic cardiac function [29,30].

## 3. Intracellular Renin Angiotensin System

The existence of an intracellular RAS (iRAS) has been studied for several years. Fibroblasts, endothelial cells, kidney cells and cardiac cells have been shown to synthesize intracellular Ang II, which has physiological effects on nuclear expression, extracellular matrix conformation, cell proliferation and vascular contraction [15]. Intracellular Ang II also stimulates nuclear AT1R and AT2R in cardiac fibroblasts, promoting the generation of NO and an increase in the calcium-dependent inositol triphosphate receptor (IP3R), modulating cell proliferation, mRNA and collagen production [31]. Extracellular ARBs do not interact with intracellular Ang II receptors [31,32]. Overstimulation of extracellular RAS in pathological processes increases intracellular RAS activity, leading to the stimulation of cardiac hypertrophy, apoptosis, oxidative stress and the increased expression of NF-κB and transforming growth factor β, thus leading to organ damage [33,34].

### 3.1. Mitochondrial iRAS

Among the organelles in which various RAS components have been described, one of the most relevant and studied is the presence of the different G-protein-coupled Ang II receptors in the mitochondrial membrane. These receptors are not encoded directly by mitochondrial DNA, but they come from the plasma membrane or can be found in the cytosol. Angiotensin II intracellular receptors have been identified primarily in mitochondria isolated from adrenal cortex cells [35] but they have also been observed in other cells [36], such as cardiomyocytes [11,36]. Protein kinase C epsilon is a protein responsible for the translocation of AT1R and AT2R from the plasma membrane to the mitochondrial membrane, as well as the activation of cardioprotective pathways such as p38, ERK 1/2, JNK and Akt [8]. This has been proven by the use of chelerythrine, a selective PKC blocker, which inhibits the activation of PKCε and blocks the translocation of the components mentioned above [37].

#### 3.1.1. Mitochondrial AT1R

It has been demonstrated that the activation of the mitochondrial AT1R stimulates NADPH oxidase 4 (NOX4) [36], which increases the superoxide concentration within the mitochondria, accelerating mitochondrial respiration. By creating high concentrations of Ang II and by using an AT2R antagonist (PD123319), researchers have obtained evidence of the activation of oxidative phosphorylation, the maximum respiratory rate and ROS production [36].

#### 3.1.2. Mitochondrial AT2R

Angiotensin II type 2 receptor is abundant in the mitochondria of non-ischemic cells, where the AT2R/AT1R ratio is higher [11]. During ischemia, this ratio is reduced, but it rises again if an APC before global ischemia is performed. This happens because Ang II promotes oxidative stress, through the induction of a compensatory increase in AT2R in the mitochondria. This mechanism has been observed in neuronal cells where oxidative stress was induced [38]. This receptor in the mitochondria activates mitochondrial NOS via the G-protein receptor pathway, which leads to an increase in the levels of NO within the mitochondria. This NO increase counteracts the effects of the superoxide produced by NOX4 and therefore reduces mitochondrial respiration [38]. This mechanism suggests that whenever a reperfusion is performed, the reduction in mitochondrial respiration would reduce the energy and oxygen demands of the heart, also reducing the production of ROS by the electron transport chain (ETC) and, thus, activating cardioprotection pathways such as hypoxia-inducible factor (HIF) and Nrf2 [5], dependent on PKCε, or via the NO/cGMP pathway [8].

### 3.2. Nuclear iRAS

Several studies have demonstrated that Ang II receptors can be present in the nuclei of multiple cells, including cardiomyocytes [38]. Following the binding of Ang II with AT1R, the receptor is translocated to the nucleus. The transport of AT2R has less evidence, but it is believed that it could be transported to the nucleus via active transport [39]. The main function of these receptors in the nucleus is believed to be related to an amplification of the response generated by the activation of Ang II receptors in the plasma membrane. The activation of nuclear AT1R would generate an increase in intranuclear superoxide via NOX4 activation [8]. If this increase in superoxide occurs in low amounts, it can stimulate the gene transcription of antioxidant substances in the same way as occurs with the moderate activation of the AT1R of the plasma membrane when APC is performed [38]. An increase in the superoxide concentration also promotes an increase in nuclear Ca^2+^ [40]. This increase is due to the activation of several transcription factors, such as DREAM, which increases the transcription of the mRNA of proteins that counter-modulate AT1R activity, such as IGF-1, AT2R and PGC-1α, leading to a reduction in mitochondrial respiration and therefore a reduction in ROS production [38]. For example, IGF-1 and PGC-1α interact with SIRT-1, thus increasing mitochondrial protection and reducing ROS. However, superoxide also increases the expression of prorenin, renin and angiotensinogen mRNA, which leads to increased intracellular Ang II levels [38]. High levels of intracellular Ang II are also associated with nuclear damage. Therefore, this apparently cardioprotective action of nuclear AT1R would only be generated in low to moderate amounts, which has not yet been determined. 

**Figure 1:**
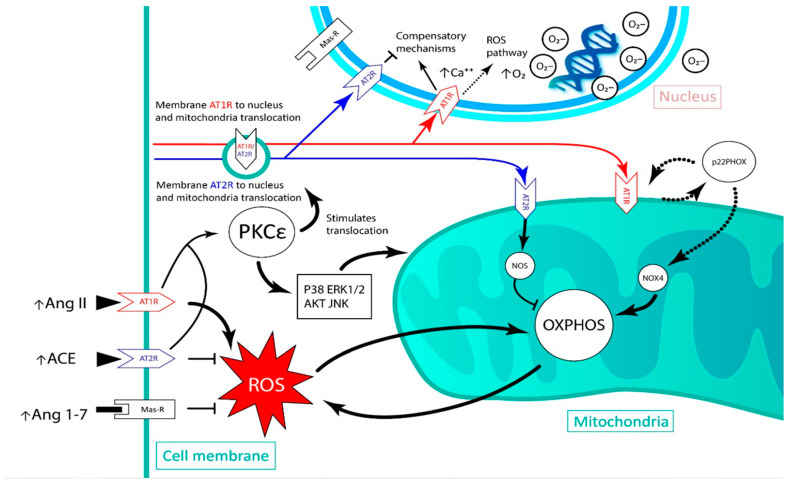
Schematic of the renin angiotensin system-induced reactive oxygen species generation pathway in the cardiac tissue after an ischemic event, and the movements of angiotensin receptors between the nucleus, plasma membrane and mitochondria. AT1R: angiotensin II type 1 receptor; AT2R: angiotensin II type 2 receptor; Ang II: angiotensin II; ACE: angiotensin I converting enzyme; Ang 1–7: Angiotensin 1–7; PKCε: protein kinase C epsilon; NOX4: NADPH oxidase 4; NOS: nitric oxide synthase; OXPHOS: oxidative phosphorylation; ROS: reactive oxygen species; AKT: protein kinase B; JNK: c-Jun N-terminal kinases.

Meanwhile, the activation of nuclear AT2R and MasR leads to an increase in NO levels, which would regulate AT1R [38]. Interestingly, the activation of the nuclear MasR, without activating AT2R, leads to a significant decrease in the expression of AT2R mRNA, but not of MasR, suggesting a type of regulation between AT2R and this receptor [38]. Given this evidence, blocking AT1R, which would be overexpressed in an ischemia event, could be expected to contribute to the increased generation of ROS and inflammation in affected cells. This could be a pharmacological target that would not only reduce the direct effect of the Ang II/AT1R axis but would also promote cardioprotective axes such as the Ang II/AT2R axis. In this way, this latter axis could exert a synergistic action with other drugs that have different pharmacological targets but share common signaling pathways, such as akt, MAPK and PKCε. The previously described pathophysiological mechanisms have been outlined in Figure 1. Paracrine and intracellular RAS and its oxidative effects in the different compartments of interest are shown.

## 4. Antioxidants

The body is continually in a state of balance between oxidants and antioxidants to maintain cellular homeostasis and prevent any amount of free radicals from becoming harmful. Free radicals are molecules that have an unpaired electron, thus leading to a condition of instability. Antioxidant substances donate electrons so that the balance between oxidants and antioxidants is maintained. Through this, the preservation of ROS levels is achieved, guaranteeing redox signaling events and at the same time counteracting oxidative damage. The antioxidant defense system works through both enzymes and other non-enzymatic molecules. Superoxide dismutase (SOD), catalase (CAT), peroxiredoxins (PRXs), thioredoxins (TRXs), glutaredoxins (GRXs) and glutathione peroxidases (GPXs) are the main enzymes and the first line of defense against oxidative damage [41]. Non-enzymatic antioxidant molecules are represented both by endogenous and exogenous components. The ones considered in this review are ascorbic acid, NAC and deferoxamine, because it has been reported that the combination of these three cardioprotective drugs could result in a synergistic effect [41]. Their biological actions are summarized below.

### 4.1. Ascorbic Acid

Ascorbic acid, or vitamin C (Vit C), is an essential, water-soluble antioxidant whose main action is to act directly to reduce ROS via scavenger actions [4]. It can be found in two states depending on its oxidation state: ascorbic acid when it is reduced and dehydroascorbic acid when oxidized [42]. Its antioxidant power is based on its ability to donate electrons, resulting in the production of dehydroascorbic acid [41]. By donating electrons, ascorbic acid produces an ascorbyl radical, which is capable of being oxidized, working as an enzymatic cofactor or an antioxidant [43].

Normal plasma Vit C levels are around 50–70 μmol/L [44]; however, Vit C must reach a plasma concentration of 10 mmol/L to displace the reaction of the superoxide anion radical with nitric oxide, which acts at a rate 105 times greater than that of the reaction between ascorbic acid and the superoxide anion radical [45,46].

In addition to its direct ROS-scavenging action, Vit C exerts an indirect antioxidant action through the modulation of certain enzymes, leading to a decrease in ROS formation. Within these modulations, Vit C causes the downregulation of the activity of enzyme NOX, which is present in the endothelium, leukocytes and myocardium, being responsible for producing superoxide. Vitamin C can also inhibit the activation of the NF-κB pathway, thus modulating the formation of cytokines that amplify the inflammatory response that normally promotes the arrival of more leukocytes and thus increases ROS formation and damage when this pathway is active. Vitamin C also prevents the uncoupling of the eNOS enzyme by stabilizing tetrahydrobiopterin (BH4), along with preventing its oxidation [41].

Likewise, it has been seen that ascorbic acid can modulate endothelial function [39], reducing the expression of NADPH oxidases and overexpressing antioxidant enzymes, particularly superoxide dismutase (SOD) and eNOS through the prevention of dihydrobiopterin oxidation and inhibiting the expression of the p47 PHOX subunit, as well as the overexpression of phospholipase A2 [47].

### 4.2. Deferoxamine

Iron ions play a major role in ROS production [48] on a myocardial reperfusion insult, as, in this phenomenon, the intracellular iron concentration rises as a consequence of ferritin saturation [49]. Free intracellular iron ions participate in the Fenton reaction and the Haber-Weiss reaction, the first being a chemical reaction between ferrous iron and H_2_O_2_, producing hydroxyl radical by oxidizing iron; in the latter, ferric iron is reduced again in the presence of superoxide radicals, also producing hydroxyl radicals [50].

Deferoxamine (DFO) acts as an extracellular iron chelator and has a great affinity for Fe^3+^, providing its principal inhibitory effect on hydroxyl radical generation, forming a stable complex with Fe3+ and decreasing its availability for ROS production [51]. It was described that, upon ischemia–reperfusion injury onset, it improved cardiac function in rat models [52].

Ascorbic acid reduces Fe^3+^ to Fe^2+^, a substrate used in the Fenton reaction; therefore, adding an iron chelator as DFO could create a synergistic effect, enhancing Vit C’s pro-oxidant effects [53].

### 4.3. N-Acetylcysteine

N-acetylcysteine is a GSH donor that has a synergistic effect with ascorbic acid, because the latter consumes GSH to exert its antioxidant action [4]. This drug could prevent the reduction of the GSH/GSSG ratio, by the previously mentioned mechanism. The relevance of this effect is expressed in the reperfusion phase of an AMI due to ROS burst, which can be controlled by stabilizing the GSH/GSSG ratio by the addition of ascorbic acid [53]. Moreover, NAC has an indirect action as a metal ion chelator and an ROS-scavenging effect, along with the ability to inhibit NF-κB [54].

## 5. Discussion

**Figure 2:**
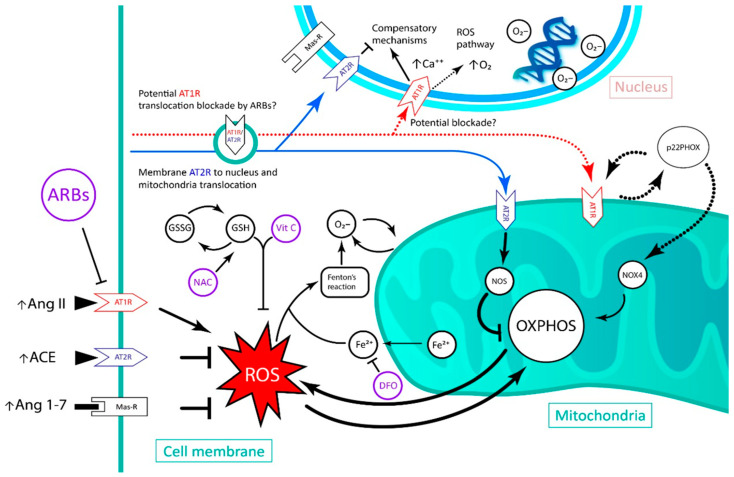
Schematic of the pharmacological effects of deferoxamine, ascorbic acid and N-acetylcysteine in the injured cardiomyocyte, with the possible benefits of adding an angiotensin receptor blocker. ARB: angiotensin receptor blocker; NAC: N-acetylcysteine; Vit C: vitamin C; GSH: glutathione; GSSG: glutathione disulfide; DFO: deferoxamine; AT1R: angiotensin II type 1 receptor; AT2R: angiotensin II type 2 receptor; Ang II: angiotensin II; ACE: angiotensin I converting enzyme; Ang 1–7: Angiotensin 1–7; PKCε: protein kinase C epsilon; NOX4: NADPH oxidase 4; NOS: nitric oxide synthase; OXPHOS: oxidative phosphorylation; ROS: reactive oxygen species; AKT: protein kinase B; JNK: c-Jun N-terminal kinases.

With the data presented, it seems that the clinical trials that have proven a cardioprotective role during APC are those where a mild to moderate amount of Ang II was present before ischemia induction [11]. Conversely, with our review, we aimed to present data that validate the use of ARBs during ischemia, and especially before reperfusion, considering that, in AMI patients, reperfusion therapy causes heart damage to largely increase in size. The aim of this therapy is the reduction of the deleterious effect of Ang II in increasing ROS and in producing cellular damage, since the increase in intracellular concentrations of Ang II in acute form generates organelle damage through the activation of AT1R, due to an increase in superoxide production [9]. It has been described in rat models of IRI that this insult induces an increase in AT1R expression, and, in contrast, AT2R expression decreases [55]. Given these results, ARBs can be considered useful to target and reduce reperfusion damage in humans. Indeed, it has been described in animal models that in the ischemic–reperfused heart, AT1R blockade increases the expression of AT2R in the infarcted area [55,56] and causes a coronary flow improvement after reperfusion [57]. A proposed model of this interaction is presented in Figure 2. 

Kim et al. [58] described that patients that were treated with losartan early after the onset of an AMI had a lower incidence of ventricular late potentials, lowering the risk of arrhythmia in these patients. In addition to this, multiple isolated therapies have been experimentally tested, with positive results in reducing mortality and the size of the infarcted area, but when they were tested in clinical studies, the results were controversial or null [59]. Recently, it has been strongly suggested that the use of multitarget therapies may be beneficial. Multitarget therapy is defined as the simultaneous use of various drugs with known targets to block the pathophysiological mechanisms of reperfusion so that an additive or synergistic effect is generated [59]. The problem resides in the fact that experimental studies in animals do not reflect the clinical reality, where affected patients suffer from multiple comorbidities affecting the heart tissue. This clinical scenario is what generates ischemia–reperfusion injury [59]. 

This is particularly true in the setting of AMI; thus, the use of ARBs applied in conjunction with other antioxidant drugs may create synergistic or additive effects.

The use of ARBs combined with tritherapy, as proposed by Rodrigo et al. [41], would have a synergistic antioxidant effect and could lead to the greater prevention of reperfusion damage in an infarcted heart by synergistically or additively reducing ROS levels and inflammation, the main agents in the production of reperfusion damage. This would occur due to the reduction of mitochondrial dysfunction, NADPH oxidases’ action, the overexpression of antioxidant enzymes and the reduction of abnormal mitochondrial respiration due to the predominant action of AT2R.

Thus, while AT1R blockade reduces the activation of NADPH oxidases and the mitochondrial respiratory activity, ascorbic acid would reduce the amount of superoxide ions and the activity of NADPH oxidases [5]. Due to this common target, there could be an additive or synergistic effect. The application of NAC in dopaminergic neuronal cells leads to an increase in nuclear AT1R expression, via the reduction of nuclear calcium and superoxide anions, thus counter-regulating AT2R expression [36]. As cardiac cells share both angiotensin receptor pathways, we suggest that the use of ARBs could decrease this adverse effect. However, clinical studies that evaluate whether the above-mentioned would occur are still lacking.

A vast portion of the RAS pathway has been shown to be stimulated in the infarcted heart [60,61,62], and especially the paracrine RAS system in the infarcted area [63]. Within these components, the role of angiotensin-converting enzyme 2 in cutting Ang II has been described for the generation of Ang 1–7 [64,65,66]. Therefore, it can be assumed that Ang 1–7 levels increase in the infarcted area, stimulating the activation of the MasR [67]. Indeed, Upadhyay et al. described how Ang 1–7 potentiates the cardioprotective activity of IPC in ischemia–reperfusion-challenged rats, both in direct and chronic sets of experiments. This study also highlighted the role of Ang 1–7 in attenuating mitochondrial dysfunction, oxidative stress, and mitochondria-dependent apoptosis in ischemic–reperfused rats. Furthermore, some studies regarding Angiotensin 1–9’s effects on the heart have been conducted. Specifically, Mendoza-Torres et al. [68] demonstrated that administering Angiotensin 1–9 at the time of reperfusion reduces the infarct size and preserves left ventricular function in isolated perfused rat hearts subjected to acute I/R injury, through a mechanism dependent on both AT2R and Akt.

In conclusion, the present review suggests the combinatory use of AT1R blockers and antioxidant cardioprotective tri-associated therapy (Vit C, NAC and DFO). The aim of this multitherapy is to inhibit the activation of the Ang II/AT1R axis and the associated ROS burst, and to further enhance the findings of decreased IRI in the infarcted heart. The synergistic effects mentioned regarding this association must be further investigated, and we encourage the development of preclinical trials to test this hypothesis.
